# Family health climate: a qualitative exploration of everyday family life and health

**DOI:** 10.1186/s12889-021-11297-4

**Published:** 2021-06-29

**Authors:** Hagen Wäsche, Christina Niermann, Jelena Bezold, Alexander Woll

**Affiliations:** 1 Institute of Sports and Sports Science, Karlsruhe Institute of Technology, Karlsruhe, Germany; 2 Department of Sport Science, University of Konstanz, Konstanz, Germany

**Keywords:** Family, Family health climate, Health behavior, Physical activity, Eating, Qualitative research, Interview

## Abstract

**Background:**

The family is an important social environment for children’s, adolescents’ and adults’ health. However, studies mostly focused on dyadic and unidirectional influences of parents on their children. Studies addressing influences arising from daily family life and including family-level influences are rare and the existing studies solely focus on the relevance for children’s health or health-related behaviors. We use a qualitative approach to explore how daily family life and its inherent health-related cues affect family members’ physical activity and eating behavior.

**Methods:**

Semi-structured interviews utilizing an interview guide were conducted. Since we aimed to examine family life, we analyzed both parents’ and their children’s views on health-related interaction patterns and family environmental influences on individuals’ health-related behavior. Twenty-two members of seven families were interviewed. Transcripts of the interviews were systematically analyzed following Grounded Theory principles.

**Results:**

The interviews revealed that various individual as well as environmental factors shape health-related aspects of daily family life. A model was developed that organizes these influencing factors on family life with regard to health-related interactions and the emergence of the Family Health Climate (FHC) – reflecting shared perceptions and cognitions regarding a healthy lifestyle within families – and its consequences. Family interactions and family time, often realized through shared family meals, are key factors for families’ health with regard to nutrition and physical activity. The FHC showed to affect various aspects related to health behavior of individual family members.

**Conclusions:**

The model sheds light on underlying processes and mechanisms of family life that influences individuals’ health-related behavior. Based on a better understanding of the association between family life and individual health behavior the development of family-based interventions can be informed. Furthermore, the insights can help to guide further research focusing on families as a system.

## Background

Individuals’ health behaviors are embedded in social contexts and affected by social ties [1, 2]. According to socio-ecological perspectives individual’s behavior can only be understood by taking the social and physical environment into account. One of the most important social contexts is the family. Daily family life implies a large number of health-related cues, such as family meals, choice and preparation of food, (in)active commuting, joint physical activities and communication about health-related issues. Specific formal or informal rules may develop, which stimulate individuals’ health behavior patterns [1, 3]. Internalized concepts of health and well-being, values, attitudes and self-perception of competences are formed within the family [4, 5]. Furthermore, the family is an instance of control and provides socio-emotional support [6].

The relevance of the family for children’s and adolescents’ health related behaviors has been shown in many studies. The majority of these studies have focused on the parent-child dyad and examined the influence of parents on their children [7, 8]. However, according to theoretical approaches such as Family Systems Theories it could be assumed that there are family-level socialization dynamics [9] beyond dyadic parent-child interactions that affect the development and maintenance of a healthy lifestyle of all family members. Families are complex interacting systems and organized wholes [10, 11], based on relational processes or reciprocal interactions among family members. Families are social networks with emergent characteristics that cannot be explained solely by individual attributes [12]. To analyze and understand such networks, relational structures among family members (e.g., dyads, triads, tetrads etc.) have to be considered. These relations enable reciprocal communication, coordination, support or trust building. From a network point of view, family structures are not only a result of the interaction of each family member. The structures, in turn, do also influence each family member [12].

Few studies have engaged with family-level socialization dynamics described in Family as System Theories and addressed family-level factors with regard to individual’s health. An example is family functioning, which comprises structural and organizational properties of the family and its interpersonal interactions. Family functioning is reflected in aspects such as communication patterns, role fulfillment, adaptability, management of conflicts, involvement, warmth/closeness, and behavior control. Studies found that family functioning is an important correlate of health-related behaviors in children [13–15]. A further example for a family-level factor is the Family Health Climate (FHC) [16]. This concept is based on the idea that individuals within a family interact with and reciprocally influence each other. These interactions take place over an extended time period and with a high frequency and constitute a ‘climate’ representing an essential component of family members interrelationship and the family environment. The FHC reflects shared perceptions and cognitions regarding a healthy lifestyle within families. It is reflected in the evaluation of health-related topics within the family and in expectations regarding typical values, behavior routines and interaction patterns. The FHC serves as a framework for individual’s health-related behavior in daily life. From a systems point of view, it is the basis of regulating health-related behaviors and provides references for valuing and interpreting the own behavior and that of others. Hence, the Family Health Climate is an aspect of the family environment that shapes the daily health behaviors of the family members, both within and outside of the family [17, 18].

In sum, the family is an important social environment for children’s as well as parents’ health. However, studies addressing family-level influences are rare and the existing studies solely focus on the relevance for children’s health or health-related behaviors. To the best of our knowledge, there are no studies that addressed children’s and parents’ health behaviors simultaneously.

Therefore, we aim to include both parents’ and children’s views on health-related interactions within the family and family environmental influences on individuals’ health-related behavioral patterns. For our analysis, we take the concept of FHC as a starting point and focus on its antecedents and consequences. To explore how daily family life and its inherent health-related cues affect family members’ physical activity and eating behavior, we use a qualitative approach. Gaining knowledge on factors influencing aspects of daily family life that are related to health-related consequences (e.g. behavior, attitudes) for the individuals’ and examining the underlying processes and mechanisms, is crucial to inform the development of family-based interventions and to further develop theoretical approaches.

### Objectives

The aim of this study is twofold:

1. To identify key health-related factors that affect daily family life and to explore how they are related to individuals’ health-related behaviors.
2. To organize these factors and their relations within a theoretical framework explaining antecedents and consequences of the Family Health Climate as a core aspect of this study.

## Methods

### Procedure and participants

The interviews took place between February and May 2016 in the region of Karlsruhe, a city in the southwest of Germany. We applied purposeful sampling to identify and select information-rich cases, that is, families that have knowledge and experience about the phenomenon of interest, in an effective way [19]. Participants were recruited in schools via distributing flyers and posters after directors’ agreement and by placing a project description in an university-internal magazine (KIT-Dialog). In addition, flyers were distributed in regional sports clubs. The advertising material, where the families are addressed as “experts of daily family life”, included brief information about the aims of the interview study, that is, to explore how daily family life shapes individual’s physical activity and eating. Furthermore, the inclusion criteria (having at least one child between 10 and 16 years) was described and contact information was provided.

Families with at least one child aged 10 to 16 years and one parent were included in the study. There were no further inclusion or exclusion criteria. Interested families were contacted by study staff who informed them about content, aims, methods, and the procedure of the study.

If families gave verbal consent, a date for the interview was set. Prior to the interviews, participants got written information about the background, aims and the procedure of the study. Additionally, each family member was informed that their participation in the study was voluntary and that it is possible to opt out at any time. Finally, each family member signed an informed consent. Through our sampling strategy, seven families, comprising 22 individuals, expressed their interest to share their knowledge and experiences with us and were interviewed.

The study was conducted with ethics approval from the Karlsruhe Institute of Technology Research Ethics Committee.

### Data collection

Since we aimed to examine family life, we did not interview single members but the whole family at once. Interviewing the whole family provides better insights in their living environment, interaction patterns and experiences within the family [20]. Therefore, semi-structured interviews utilizing an interview guide were conducted. The interview guide was developed by a team of three researchers in an iterative process including feedback loops enriched by discussions with parents. The interview guide focused on daily family life and families were encouraged to create links to physical activity and eating behavior. The guide comprised four topics with three to four questions per topic. First, in the introductory part, some general questions were asked (e.g., about age, job, working hours, school). Furthermore, we asked about the amount and type of time spent together as a family. In a second part, the questions dealt with aspects concerning family health, e. g. the importance of a healthy lifestyle in the family, specific health related actions or disagreements concerning health. Participants were also asked to recall and describe specific situations in which these phenomena appeared. Third, the families were asked about their opinion on typically healthy or typically unhealthy families. In this context, participants were asked about the health status of their own family, potential for improvement and barriers they experienced. Up to this point, questions were not specifically related to nutrition or physical activity. Only the last topic comprised questions about similarities and differences directly related to physical activity and eating behavior in the family. We also asked for reciprocal influence on physical activity and eating behavior. Interviewers received special training prior to the study, with information on possible difficulties in a family setting and how to work with back up questions (e.g. providing examples) when the interview comes to a halt. Each interviewer conducted at least one pretest interview with feedback by the researcher team. The interviews took place at the participants’ homes in order to conduct the interviews under favourable environmental conditions [20, 21]. To avoid a parent-dominated conversation, we encouraged every family member to answer the questions and have not set a time-limit for the interviews. All interviews were held in German.

### Data analysis

The interviews took between 55 and 85 min, were digitally recorded and transcribed verbatim. Transcription and data analysis were conducted using F4Analysis (dr. drehsing & pehl GmbH), a software package for qualitative analysis. Transcripts were systematically analyzed following Grounded Theory principles [22]. Open, axial and selective coding was applied to all transcripts. Firstly, raw text data was coded and compared in order to categorize relevant information with similar meaning. While reading and coding the manuscripts, ideas were written down in memos to support the process of open, axial and selective coding. In a second step, connections between the categories within and between cases were established and checked in a circular process. Finally, selective coding involved the identification of core categories and their connections. For data analysis two researchers read and analyzed the interviews and weekly meetings were held to discuss the findings and to develop a theoretical model according to Strauss’ coding paradigm [23]. If needed, a third researcher was consulted to solve discrepancies between the two coders. In this process, previous theoretical knowledge and sensitizing concepts were utilized to construct empirically grounded categories [24], focusing on the Family Health Climate. For presentation in this paper, quotations were translated from German to English.

## Findings

All of the seven interviewed families were two-parent families. At least three family members (two parents and at least one child; three families) participated in the interviews. Three interviews were conducted with four family-members and one interview with five family members. In three families all family members were interviewed, while in the other families, children who were younger than 10 or older than 16 years were not included in the interview.

Fathers’ (*n* = 7) mean age was 47 years (SD = 4.4, range = 39–51). Mothers’ (n = 7) mean age was 44 years (SD = 3.7, range = 38–50) and children’s (*n* = 8) mean age was 13 years (SD = 2.1, range = 10–16). We asked the participants to estimate their average weekly working and school hours as an indicator of the available amount of potential family time per person and week. On average, fathers spent about 42.9 (SD = 7.3) hours per week at work and had therefore the least family time. The mothers spent less time at work, on average 22.9 h per week (SD = 7.4), and had therefore more time together with the children at home, which spent on average 32.2 h per week (SD = 2.9) in school. Therefore, on average shared family time occurs most of all in the afternoons (given that school takes place and part-time jobs are usually done in the mornings) and evenings on weekdays and on the weekends. These findings represent the traditional family structure and division of roles in German families.

**Fig. 1:**
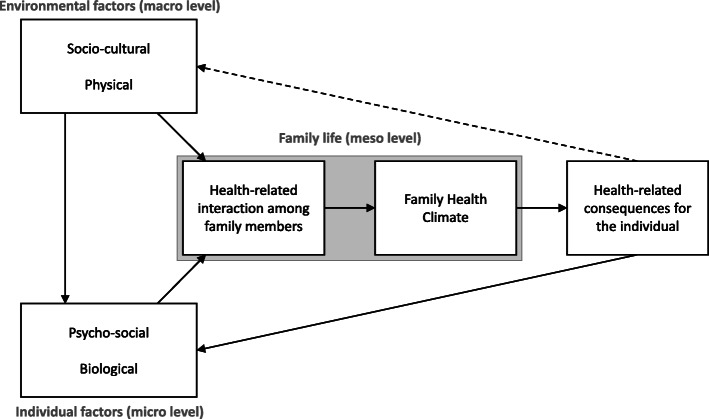
Influential factors and health-related consequences of daily family life

We identified various factors arising from the socio-cultural and physical environment as well as individual factors that affect family life as reflected in health-related behaviors and interactions. Based on these findings, we developed a model of factors shaping the Family Health Climate (FHC) and consequences of the FHC. It provides a comprehensive understanding of relevant elements and their associations with regard to the FHC. As such, the model goes beyond the concept of FHC, which was used as a starting point for this study. For a better understanding, we present the model at the beginning of the findings section (see Fig. 1). Subsequently we discuss the findings with regard to the elements and their associations of the model.

The central element of the model is the family life (meso level). With regard to health, the family life comprises health-related interactions among family members that result in the Family Health Climate. These health-related interactions among family members are based on various actions of individual family members that refer to other family members. These actions are influenced by individual factors such as personal attitudes and interests, genetic predispositions, motor competences etc. However, individual behavior is also influenced by socio-cultural norms, the community and the neighborhood, workplaces and schools the individuals of a family are embedded in. Hence, actions of individual family members are affected by individual factors at the micro level and environmental factors at the macro level. While individuals’ actions are based on individual intentions (i.e. one-sided), interactions reflect reciprocal actions in dyads (i.e. two-sided) or triads etc. The sum of interactions forms the network structure of families at the meso level. Recurring family interactions such as health-related information exchange or shared activities (e.g., accompanying children to competitions) and family routines (e.g., eating dinner together) result in shared cognitions and values as reflected in the Family Health Climate. These shared cognitions and values, in turn, have consequences for the health of the individual. They can influence and change cognitive, motivational and behavioral factors with regard to health and may foster a healthy or unhealthy lifestyle. This lifestyle can affect biological aspects such as Body Mass Index (BMI) or individual attitudes and interests and eventually causes circular closure of the model. It should be noted, however, that attitudes and behavior of many individuals can eventually contribute to the reproduction or changes of socio-cultural norms or changes of the built environment at the macro-level. However, these effects are not straightforward and subject to other environmental influences such as community policies and economic dynamics. With our study design we are not able to analyze this association, which is why the association is depicted by a dashed arrow. In sum, the model comprises our main findings and describes a circular process that enables a better understanding of the Family Health Climate and its effect on individual health.

In the following, the main categories of the model – family interactions and Family Health Climate – and their relation to the other elements of the model are described and discussed based on the analyzed interviews.

### Influences on health-related interactions among family members

Health-related interactions among family members are affected by individual and environmental factors. First, we discuss biological and psycho-social factors (individual), followed by factors of the socio-cultural and physical environment (environment).

#### Biological factors

The interviews revealed that biological factors affect both individual’s own health behaviors and the health behaviors of other family members. With regard to biological factors, the age of the children is a salient example. When children are young, family life is often associated with a lack of time for parents’ physical activities, especially for mothers.

*Well, for a really long time I did nothing [with regard to sports]. I was absolutely busy with the children, and the family, and this and that. I had no more energy. It was much harder to leave the house. If you can’t leave the children alone, it is hard to organize things and to say ‘Now I go for sports’. It’s very hard to integrate it. (Mother 4)*

Another challenge were age and gender differences of the children in a family that make it difficult to do sports together:

*Well, this is the challenge. Since [name of 1*^*st*^ *child] is 7 years old, [name of 2*^*nd*^ *child] is 13, and [name of 3*^*rd*^ *child] is 11 – boys and girls. It’s sometimes hard to find something you can do together. (Father 2)*

Existing food intolerances of family members likely result in a more conscious handling of family nutrition. This handling is reflected in adapted family meals that affect the eating behavior of the other family members:

*I have a special position in the family. When we prepare meals with milk, we often take lactose-free milk. The family is very considerate of me. And this is very good for me. I feel much healthier than one and half year ago. (Father 7)*

In the interviews it became clear that biological factors such as age, gender and food intolerances are significantly affecting health-related interactions among family members.

#### Psycho-social factors

Psycho-social factors such as individual preferences, attitudes, beliefs or values regarding foods and physical activities affect individuals’ health behaviors and influence daily family life. One example for such an influence was a vegetarian (exclusion of animal meat from diet) or vegan (exclusion of all animal products such as meat, dairy, eggs) lifestyle:

*I was vegetarian for six and a half years and now I am – since a few months – vegan and that is something essential for our diet [of the family]. (Child 3)*

A vegan diet affects joint actions in the family such as grocery shopping, meal planning, and family meals and might also affect the eating behavior of other family members.

In general, planning of family meals was reported to be difficult. It depends on different food preferences, tolerances or taste and some parents complained about the difficulties caused by various wishes:

*So, the whole planning, what we eat and that already begins at noon. What do we eat today? I already know who has which needs and try to consider all of them to some extent, which is sometimes not that easy. (Mother 4)*

Most of the families reported that individual food preferences were associated with difficulties and sometimes led to conflicts. While in these cases food preferences of individual family members influenced food-related family interactions, there were also consequences of these interactions that were considered to be challenging, e.g. for the mother:

*Well, sometimes when making the meal plan I found it exhausting and I thought, ‘No, what am I doing here? What an effort!’. So, it’s like a conflict with myself, where I’m thinking: ‘Shit, what have I done wrong?’.* (Mother 2)

Obviously, different needs with regard to meals can, in turn, affect individual’s psycho-social factors such as perceptions of stress. In the cited cases, stress was a consequence of individual preferences within the family that were not part of shared cognitions about family meals. This is a noteworthy example because it shows that family interactions can also have negative consequences for the individual.

A similar pattern was apparent with regard to physical activity. Different activity preferences and motivational levels made joint physical activities a challenge. For example, parents wanted to go hiking or walking whereas their children prefered other activities:

*Sure, this brings the mood down. When I say ‚Come on, let’s go hiking at the weekend!‘ then one or two [children] say ‚no way‘. (Mother 2)*

*We are simply individuals, and it is really a challenge to find a common thread, also with regard to physical activity. (Mother 2)*

The interviews revealed that individual preferences and values have a significant impact on health-related family interactions. Distinct preferences often lead to difficulties and conflicts in the family. In sum, psycho-social and biological factors influence health-related interactions in several ways.

#### Socio-cultural factors

Various socio-cultural aspects affect health-related interactions among family members. It should be noted that many of the socio-cultural factors have an immediate influence on individual family members and do not directly affect family interaction. Nevertheless, the resulting actions were uttered by the interviewees with regard to the family context. As such, these socio-cultural influences exert an indirect influence on family interactions.

In the interviews, parents described today’s social lifestyle as fast moving, hectic and full of time pressure, causing a general lack of time. As a result, various families reported that a lack of time hinders conducive health-related interactions among family members, e.g. talking about healthy eating or discussing the next family meal. The lack of family communication about health-related issues affects shared values such as healthy eating or eating together as a family. They are less stable, which can result in less frequent family meals or unhealthy eating routines. In turn, this has a negative impact on individuals’ health behavior.

*I think the critical issue is time. We live in an extremely fast-moving time, in an extremely hectic time . . . And if you are not able to structure your daily life and if you live from hand to mouth, it doesn’t work out, not at all. Then, you normally subsist on junkfood, well, such things like MCDonalds, kebab, Chinese snack or just, I would just say, thawing some frozen food and eat it. Because there is a lack of time. (Father 6)*

Furthermore, social actors showed to be relevant. In many instances, peers are an important factor of the socio-cultural environment affecting individual’s health behavior and subsequently family life. In the interviews, various children reported that their health behavior is affected by their friends, e.g. with regard to picking up a sport:

*Basketball then came through my classmates, well, a friend of my class also played. I always found that interesting, too. And then I went along once, then I had fun and so I stuck to it. (Child 4).*

With regard to eating behavior, one father uttered his surprise on how his son changed his attitude towards a healthy meal (i.e. salad) during a sports training camp with friends:

*The boys went to the handball training camp a few times now (…). And there the kids also eat together. You can’t believe what happens there. Our son came home in the evening and said: ‘I ate salad’. (Father 6)*

As a consequence of the peer influence as described in this case, family meals can include more healthy options that are accepted by the children.

While peer influence was often reported to be positive, it can also be negative, e.g. when it comes to comparisons and ‘competitions’ of being skinny and eating excessively healthy:

*Yes, especially in my age it can be realized quite clearly. There are a few people that develop an eating disorder, for sure. In my age it’s a big issue. (Child 4)*

Here, a daughter reported how social norms among female adolescents can have a negative influence on health behavior. This negative influence, in turn, affected family interactions, as the mother of the child remarked:

*We three girls [mother and two daughters] talk extremely often. I’m often about to say it’s enough. Sometimes you should just eat something without wondering how much fat is in it or anything else. You can just eat it. But for us it is a big issue I’d say – for me and the girls. (Mother 4)*

In this case, the mother considered the constant talk about food to be problematic and it seems as if she fears negative consequences.

In sum, the interviews revealed that socio-cultural factors trigger various health-related family interactions. However, it appears that influences are often moderated by individual family members.

#### Physical environmental factors

The interviewed families mentioned various relevant aspects of the physical environment. In contrast to socio-cultural factors, these aspects were mainly reported as factors that directly affect health-related interactions on the family level. In the following two citations it becomes clear that living in a hilly village or in a bike-friendly city can make a difference on the activity level of the whole family:

*Well, sometimes we briefly talk about if we go somewhere by bike or by car. But I think – that’s my impression – that developed great. I think this is related to the fact that going by car in the city became unbearable. Our family uses the bike for nearly all ways by now, that’s very nice (...). I like it, we just get a bit of physical activity into everyday life. (Father 7)*

*To go by bike is here in [name of the village] a bit difficult. To go downhill is easy, but to go uphill is a bit problematic here. (Mother 5)*

Moreover, accessibility of shopping facilities was mentioned as an important influencing factor for a healthy diet:

*The basic condition [for a healthy family] is that you don’t have to go that far for grocery shopping to get fresh things there. (Mother 6)*

Next to physical factors due to the built and natural environment, the interviewees reported that daily weather conditions and the climate depending on the seasons, affects individual health-related behavior and health-related actions on the family level like shared physical activity:

*It also depends on the season. (…). In the summertime we go together to the public outdoor swimming pool, or we go biking or running outdoors. (Mother 7)*

In the interviews, various situations were identified that underline the importance of the physical environment. The physical environment affected family members’ behavior and prompted family interactions immediately. Different to the socio-cultural environment, health-related behavior was mainly affected with regard to physical activity.

### Emergence and consequences of the family health climate

The Family Health Climate (FHC) is formed through mutual interactions in the family and is reflected in shared cognitions or values within a family. These shared cognitions and values might affect individuals’ behavior. Therefore, the FHC is supposed to foster or hamper individual’s health related behaviors.

#### Emergence of the family health climate

Shared time as a family turned out to be an important prerequisite for the emergence of the FHC. When the families were asked in which situations they share time together, it appeared that family meals play an important role:

*There is almost no shared time on weekdays, only during the meals which is limited and mostly late in the evening, then we sometimes watch TV together or something like this and then the day is over. (Mother 1)*

Statements clearly pointed towards the importance of family meals regarding shared family time.

*When we spend time together during the week, it’s almost only for eating. At least for breakfast and for dinner (…). Once a day we need to have a joint family meal. To see each other at least once a day.* (Mother 6).

*Yes, this is something that binds a family together. As [name of Mother 6] said, if you see each other at least once a day, you can talk to each other. (…) Health can not only be defined in terms of physical but also of mental health, i.e. talking about problems for example*. (Father 6)

Here, sharing time is an important family value realized through eating together as a daily routine. While this does not necessarily imply healthy eating, it shows that eating together as a family contributes to the emergence of the FHC.

Eating together also provides the opportunity to influence healthy eating of all family members:

*Yes, sometimes it goes well with fruits and vegetables, when you prepare it right after shopping in way that you can easily grab it. (Mother 6)*

Moreover, joint meals provide a platform for family conversations about health:

*And yes, I think the topic health… such topics are most often discussed at dinner. When we sit together talking, it often pops up. (Child 4)*

As such, the meals seem to be a critical factor for the emergence of shared values. This is reflected in the perception of family meals and the shared time as important. In all families, family meals were a fixed component in daily family life and were considered the easiest way for sitting together and talking to each other, but due to a lack of time family meals were only possible for dinner or for breakfast:

*We always have dinner together, I’d say. If possible, we also take our breakfast together. (Mother 4)*

*It’s a fixed ritual. We always take our breakfast together. (Father 4)*

Another example how the FHC emerges in daily family life are existing rules and restrictions. Mostly the parents were the initiators of rules. The rules concerned for example fruit and vegetable consumption, TV and smartphone use during meals as well as alcohol consumption. TV watching or smartphone use while eating was reported as a barrier for conversation and a disturbing factor for joint meals. Restrictions existed also for candies and sweetened beverages. Sometimes deals were made to support healthy eating as compensation for sweet consumption.

*Well, fruit and vegetables once a day is a must. There are no unlimited sweets. We have sweets every day but always a small portion. Sometimes a bit more, but as an exception. But that are our rules, which we have. We try to eat varied. (Mother 6)*

All families reported struggle and conflicts due to rules on eating, however, the families reported more healthy food consumption and less intake of candies and sweetened beverages consequently.

Interestingly, across all families, there was a lot more conversation about family meals than joint physical activities. However, joint physical activity was valued as well as expressed in wishes for more joint activity, which, however, is difficult to implement due to different preferences of family members:

*And there is a wish I have, that we take more time for some physical activity. For example, this year we made it only once to the public swimming pool. (Father 7).*

*But this is something I don’t like, to go to the public swimming pool. (Mother 7).*

*If she [Mother 7] would go swimming, then we would go as well. (Child 7).*

In all interviewed families, shared physical activities could almost only be realized at weekends:

*Yes, it’s like you said before [shared activities] are limited to holidays and weekends. And at the weekends, one plans with regard to the children and physical activities, and one tries to get things organized around it. (Father 6)*

The interviews showed that family values are mainly reflected in eating together as a family. As such family meals are an important catalyst of the FHC. However, there were also situations with regard to physical activities that showed how shared cognitions and family values emerge.

#### Consequences of the family health climate

The emergence of the FHC as a result of health-related family interactions has various consequences for individual family members. The following statement about the change of eating habits clearly illustrates this:

*And then we adapted a bit, and (…) primarily the meat consumption has been dramatically reduced in our family. (Father 4). (…) Yes and, for example, the variety in nutrition is changing. (Child 4).*

Besides consequences for the diet, the FHC also has consequences for the level of physical activity:

*Always. Exactly, always by bike (…). Taking the children somewhere and then pick them up. I make a lot of kilometers in a week. It’s surely 60 to 70 kilometers (…) The kids go to school by bike. They rarely get a bus tickets. (Mother 2)*

Another interesting result was happiness as a consequence of the FHC that comprises joint activities as a family value:

*Yes, it’s swimming. All of us being in the water, all of us on the waterslide, all of us being active. This is the sport that makes all of us happy, I’d say. (Mother 2)*

The influential power of the FHC is reflected in the following citation. A mother described that being active was a norm in her family, and then she compared it to other families. Reflecting on the power of different family values, she considered it to be hard to free yourself form unhealthy family values. Further, she uttered the assumption that only a certain educational level might help to reflect and detach from unhealthy behavior patterns of the family:

*We were active, we ran, we did various things without any limits, I don’t know it any other way. (…) I think that family background and educational background play an important role. The way you know it will be passed on to the children. (…) It is incredibly difficult to free yourself from an unhealthy family, away from these rituals, well, you can only make it if you achieve a certain educational level and then might be able to reflect on it and read about it, and then start to practice it by yourself. (Mother 7)*

In sum, the interviews revealed that the FHC is reflected in shared cognitions and values with regard to health. It influences cognitive, motivational and behavioral factors of the family members, and affects the health behavior of individual family members. This comprises eating habits as well as physical activities and sports.

Finally, the interviews reflected the approach to describe the family as a system, which implies that a family is a complex of interacting elements and the functioning of any one element in a system depends on the existence and operation of other elements in the system.

*I feel like a family is also a type of community. Different to living with roommates, but a community nonetheless, to which everyone contributes. (Mother 2)*

## Discussion

The aim of the study was to gain a better understanding of the association between daily family life and health. Therefore, we interviewed families – parents and children together – to identify processes and mechanisms that influence the health of the family and individual family members. With our approach, we considered family-level processes and structures, assuming that the family as a whole develops specific characteristics that manifest on a family level. Families can be considered closely interacting networks that develop norms, rules and routines that affect family health. For this reason, we utilized the concept of the FHC representing a family-level construct that is related to health-related norms, rules and routines. By interviewing 22 members of seven families we analyzed daily family life with regard to the emergence of the FHC and its consequences.

The first aim was to identify key factors affecting daily family life and the relation to individuals’ health-related consequences such as health behaviors. The interviews revealed individual as well as environmental factors that shape the health-related aspects of daily family life. This is reflected in health-related interactions among family members – the family network – in everyday family life.

First, various individual factors showed to be meaningful. Biological factors such as age, gender or food intolerances influence family interactions. Furthermore, psycho-social factors such as individual preferences and values concerning food and sports or physical activities affect health-related family interactions such as discussions regarding food preparation or joint activities on the weekends. The role of individual factors for parent-child and family interactions has also been described in the LIFES (Levels of Interacting Family Environmental Subsystems) approach [25]. The LIFES approach assumes that these factors are related to factors representing parent-child interactions, e.g. general parenting, parental cognitions and parenting practices and factors representing family interactions, e.g. family functioning, FHC, and family practices such as family meals. However, studies addressing the link between individuals’ psycho-social factors and interactions within the family are rare. Naisseh and colleagues [26] showed that parents’ level of self-determination regarding engagement in physical activities was associated with their support of children’s physical activity which can be seen as an interaction between parent and child. Furthermore, there are some studies showing that parents’ personality is related to their general parenting behavior (e.g. [27]). Our analysis contributes to an understanding of how individual factors of parents and children influence family network of health-related interaction.

In accordance with socio-ecological models [28] various environmental factors were identified. Socio-cultural factors (e.g., societal norms, peer influence) showed to influence family interactions concerning health-related issues. In most cases, this influence did not directly affect family interactions, but was mediated by an individual of the family. This is in line with studies that have shown that peers’ food choice affect children’s preferences and consumption of food [29]. Furthermore, peers are able to encourage children to eat primary disliked foods which we found in our interviews too. By influencing individual factors such as preferences, socio-cultural factors indirectly affect family interactions via the individual who transfers these influences into daily family life. We found this indirect link for example regarding the norm to be slim for adolescent girls, which make this an important topic for family interactions around dietary intake. The assumed direct link of environmental factors on family life was found in the interviews only for physical environmental factors with regard to active transport and joint activities. However, cultural norms such as the way families eat breakfast or dinner directly influences family interactions around eating as well. To the best of our knowledge there are no studies addressing the relevance of environmental factors for family interactions and daily family life.

The second aim was to organize the identified key factors to explain the antecedents and consequences of the Family Health Climate. The results showed that there are various situations that trigger family interaction and produce routines, shared cognitions and values about health-related issues. Through the interviews the outstanding role of joint family meals and associated time for health-related conversation became clear. Therefore, family meals might be an important factor for the formation of the FHC. There is a large amount of studies that addressed the relevance of family meals for individuals’ health and for family interactions. Having frequent family meals is associated with health benefits for children and adolescents, e.g., lower BMI and healthy dietary intake [13]. Patrick and Nicklas [30] call the family a social framework for eating and their results document a positive association between the frequency of family meals and children’s consumption of fruits and vegetables as well as a negative interrelation with the intake of soft drinks. A review has shown that family meal frequency is related to healthier dietary outcomes across lifespan [31]. Additionally, Berge and colleagues [32] found that both quality (e.g. distractions such as watching TV, smart phone use) and quantity of family meals are related to adults’ BMI. Furthermore, Utter and colleagues [33] found that frequency of family meals is related to parents’ well-being.

Family systems approaches describe naturally occurring family interactions as key factors for the emotional connection among family members and the feeling of coherence [34]. Family meals could be seen as a proxy for family interactions, they give structure, connection, and coherence to family life [32]: the way families manage family meals is indicative of overall family functioning [13]. There is strong evidence that frequency of family meals is associated with family functioning outcomes such as connectedness and cohesion [35].

However, family meals might have negative effects as well. In our interviews the families often reported factors that make shared meals a challenge. For example, different individual food preferences and working schedules and other commitments pose significant challenges. In the interviews, especially mothers mentioned that it is a burden for them. Accordingly, studies have shown that fussy or picky eating of children is associated with mealtime stress and conflict for parents and especially mothers (e.g. [36, 37]). However, in our interviews the families developed various strategies to overcome these challenges. One strategy mentioned was, for example, meal planning, including shopping and/or making cooking lists based on compromises. As all interviewed families managed to eat together at least for dinner or on weekends and therefore have a family ritual, this capacity to manage conflicts might be due to a good family functioning which has been shown to be associated with regular and frequent family meals (e.g. [35]). However, this link has not been studied yet.

In the end, the interviewed families emphasized the importance of shared meals, as eating together as a family has a value for both parents and children. Shared meals imply eating regularly and more healthy and spending time together. Furthermore, they provide a platform for talking and discussing health issues. Therefore, they should be considered a central antecedent of the FHC.

In the interviews it became obvious, that eating is an inherent part of daily family life whereas this is not the case for physical activity, although families wish being more physically active as a family. Families talked much more about nutrition than about physical activities, when talking about everyday family life and health. Regular meals are implemented in every family, at least once a day. In contrast, common family physical activities were less prominent in the interviewed families. Obviously, they are much more difficult to realize in daily family life. Our findings are in line with the findings of Thompson and colleagues [38]. They conducted semi-structured interviews with parents and found that parents perceived joint physical activity as important and mentioned several benefits such as spending time together and increased parent-child communication. However, parents also reported that due to busy lifestyles, diverse ages and interests of children and adults, engaging in physical activity together as a family is rare in daily family life. Activities performed together were rather sedentary. These results indicate that families consider joint physical activity as important and beneficial. However, while regular family meals are integrated in daily family life this is not the case for joint physical activity. Therefore, strategies are needed that promote and facilitate joint physical activity in families and these strategies should be integrated in family-based health promoting interventions.

Our results showed that shared family time, whether it is realized through joint family meals or activities, is a key factor in daily family life. Shared family time is essential for the development of the FHC, which is related to health-related consequences for family members. Eating more (un)healthy or becoming more (in)active or even becoming aware of healthy and unhealthy habits within the family network, are consequences of shared cognitions and values as integral part of the FHC.

In sum, the FHC is reflected in cohesion (e.g. joint meals as an appreciated part of daily life), values (e.g., importance of healthy eating, importance of being active together), and communication (e.g., talking about healthy food). However, our results show that different attitudes about food choices can often lead to conflicts. While consensus on healthy behavior can be considered an important element of the FHC [16], our study revealed that conflicts regarding health-related issues are part of family life as well. The management of these conflicts are crucial for individuals’ and families’ health and well-being.

The results of our study have several implications for research and practice. The proposed model could guide research focusing on the relevance of daily family life for individual’s health and health behavior. The model provides ideas for examining individual and environmental factors that affect daily family life, aspects of daily family life that are related to health-related consequences for the individual family member such as family interactions and Family Health Climate, and the underlying processes and mechanisms of family environmental influences on individual health behavior in quantitative studies. Furthermore, quantitative studies should focus on the emergence and consequences of the Family Health Climate that represents a family-level variable that is related to individuals’ health-related behavior. The relevance of examining the role of daily family life and family-level factors such as FHC for individual’s (children, adolescents and parents) health and health behaviors becomes particularly apparent against the background of the COVID-19 pandemic. It is probable that the restrictions during the lockdowns have an impact on daily family life (e.g. interactions, shared family time, joint meals and activities) and these changes might affect family members’ health and health behavior both positively and negatively. Although the collection of the data took place before the pandemic, the findings and the framework might assist the examination and understanding of family life and its impact on individual’s health during the pandemic situation.

The results highlight the relevance of shared family time. Therefore, interventions should consider the importance of shared family time, and aim to foster shared time in families, for example via family meals or joint activities, which provides a platform for exchange and communication and might facilitate the development of a health promoting family life, including a FHC supporting healthy lifestyles. Strategies especially for the integration of joint physical activity into daily family life are needed that consider the different prerequisites of all family members (e.g. age, sex, activity preferences).

### Strength and limitations

The major strength of this study is that we interviewed the family as a whole to analyze mechanisms influencing the health of families as complex interacting systems. However, due to our inclusion criteria of children being between 10 and 16 years, we did not consider children younger than 10 years, although they were present at the interviews. The reason for this was the assumption that younger children have a limited capability of reflecting health-related actions within the family. Furthermore, we only investigated the facets of health named by the families. Therefore, the main topics revealed are centered around activities and nutrition. None of the families addressed psychological health as an issue in their daily life. Another aspect that may limit the quality of the results is the limited involvement of children and adolescents during the interviews. This might be a consequence of the strategy to jointly interview parents and children. However, our approach ensured that natural interactions and communication patterns of the families were part of our data collection and analysis. Due to our recruitment strategy, only families interested in health-related topics took part. Including families with another socio-economic and cultural background would provide a more diverse perspective of daily family life and its relation to health-related behaviors and health. Furthermore, only dual-parent families were included. Future studies should include families with different family structures, such as single-parent families, separated parents.

## Conclusion

The present study emphasizes the importance of daily family life for the health of all family members. Family interactions and family time are key factors for families’ health with regard to nutrition and physical activity. The proposed model of influential factors and consequences of health-related family life describes how health-related interactions among family members are influenced by various factors and how the FHC emerges from this network of interactions. Finally, the FHC, that emerges through family interactions and family time, showed to affect various aspects related to health behavior of individual family members. The strength of our model is the systems perspective on family health as a network of reciprocal interaction resulting in the FHC. Future research can build on our model to focus on specific processes and mechanisms of health-related behavior on a family level. Furthermore, the model could inform the development of interventions aiming to promote individuals’ and families’ health by taking into account factors reflecting family interactions and family time that influence the emergence of the FHC.

## Data Availability

The datasets generated and/or analysed during the current study are not publicly available. They are available from the corresponding author on reasonable request, subject to approval from the ethics committee that approved the study.

## Abbreviations

FHC: Family Health Climate
BMI: Body Mass Index
LIFES: Levels of Interacting Family Environmental Subsystems
